# Experiences among men with localised urinary tract infection in primary care: a qualitative study

**DOI:** 10.1080/02813432.2026.2647002

**Published:** 2026-03-29

**Authors:** Mia Tyrstrup, Hedvig Gröndal, Malin André, Helena Kornfält Isberg

**Affiliations:** ^a^ Department of Clinical Sciences, Family Medicine and Community Medicine, Lund University, Malmö, Sweden; ^b^ Department of Biomedical Sciences and Veterinary Public Health, Swedish University of Agricultural Sciences (SLU), Uppsala, Sweden; ^c^ Department of Public Health and Caring Sciences, Family Medicine and Preventive Medicine, Uppsala University, Uppsala, Sweden

**Keywords:** Localised urinary tract infection, men, antibiotic resistance, primary health care, treatment guidelines, antibiotics, qualitative research

## Abstract

**Background:**

General practitioners (GPs) do not see men with localised urinary tract infection (UTI) very often which limits their possibility of developing expertise in the area. To gain knowledge of male patients’ experiences and perspectives on localised UTIs, qualitative research is needed.

**Aim:**

To explore expectations, experiences and symptoms in men with localised urinary tract infection in primary health care (PHC).

**Design and setting:**

A qualitative study based on semi-structured interviews with men with a history of localised UTI treated in PHC was performed.

**Method:**

Data were collected from patients with recent experience of localised UTI, using semi-structured interviews. An interview guide with open-ended questions was used. All interviews were audio recorded and transcribed verbatim. A thematic analysis was performed.

**Results:**

The median age of the 18 patients was 77.5 years. The interviews lasted an average of 16 min. Four themes emerged: (1) stigma and self-blame in managing illness. (2) Adaptation, careful planning and normalization. (3) Gender and help seeking behaviour. (4) Healthcare experience-uncertainty and trust. Many patients reflected on the causes behind their infection and expressed aspects of self-infliction. They explained that symptoms from the localised UTI affected their daily lives and adjusted their way of living according to them.

**Conclusions:**

Localised UTI symptoms in men affect their daily lives. GPs should be perceptive regarding any beliefs among patients with localised UTI, as well as any self-imposed guilt that could lead to unnecessary lifestyle changes. Although the patients in this study expressed good confidence in health care, they also found the care not individualized enough.

## Introduction

Urinary tract infections (UTI) in both women and men are mostly managed in primary health care (PHC). According to the European Association of Urology (EAU) guidelines [1], UTIs are categorised as either localised or systemic based on clinical signs and symptoms. A localised UTI refers to cystitis without systemic features in individuals of any sex whereas a systemic UTI involves signs and symptoms of systemic infection, with or without accompanying local urinary symptoms, and may arise from any site in the urinary tract [1]. This study focuses on localised UTIs in men.

Patients with localised UTI typically present with acute onset of symptoms such as frequent micturition, dysuria and urgency with nocturia and suprapubic discomfort also reported [2,3]. The management of localised UTI in men in PHC lacks international consensus, Swedish national guidelines recommend treatment with narrow-spectrum antibiotics for seven days [4]. localised UTI is rare in younger men but the incidence increases with age. The incidence of UTI in men aged <55 years is 0.9–2.4 cases per 1000 and up to 7.7 per 1000 in men aged ≥85 years [5,6]. In 2013, the consultation rate in Swedish PHC for localised UTI was 12 per 1000 among men versus 73 per 1000 among women [7].

Because localised UTI in men is relatively infrequent, general practitioners (GPs) have limited opportunity to develop diagnostic experience. Qualitative studies in Ireland and Sweden suggest that GPs find male localised UTI challenging to diagnose and often undertake more extensive examinations than in women [8,9]. More broadly, diagnostic uncertainty is intrinsic to general practice, especially for infrequent or non-specific presentations, contributing to variability in management [10–12].

Little is known about men’s own experiences of localised UTI in PHC, including their symptoms, concerns and expectations. Given the low prevalence of male localised UTI and the resulting limited GP experience, systematically integrating patient perspectives may be essential for improving diagnostic accuracy. Findings from this study may help support GPs in managing a condition they encounter infrequently and may ultimately improve diagnostic rigour, communication and patient centred care. We therefore conducted a qualitative study, using a patient-centered approach as advocated by multiple organizations, including the World Health Organization (WHO) [13,14] to explore male patients’ perspectives on localised UTI in PHC.

The aim of this article is to explore symptoms, expectations and experiences in men with localised UTI in PHC.

## Materials and methods

In order to explore male patients’ experiences and concerns regarding localised UTI, the study draws on a qualitative approach. Data were collected from patients with recent experience of localised UTI using semi-structured interviews [15,16]. We followed the Standards for Reporting Qualitative Research (SRQR) [17].

Managers at 23 PHCCs across rural and urban areas of Skåne, Sweden were invited to the study. Seven PHCCs participated. Using electronic medical records, PHC managers identified all men ≥18 years diagnosed with localised UTI in the previous six months (*n* = 61) and mailed invitations. Interested patients returned consent to contact forms and were then informed by phone by a researcher. Exclusion criteria were residency in nursing home or not able to understand or speak Swedish or English.

Interviews were conducted at PHCCs, at home or by telephone, with no incentive. Interviewers had no prior relationship with participants. We used a semi-structured interview guide with open-ended questions developed from prior localised UTI literature and PHC qualitative work, piloted and refined after two interviews to improve structure and flow [15,16]. The questions focused on patients’ localised UTI symptoms, their impact on daily life, accessibility to healthcare when seeking treatment due to localised UTI symptoms, expectations, fears and treatment (supplement 1).

Thirteen of the interviews were performed by a GP researcher (HKI) and five were performed by a junior doctor specializing in general medicine. Recruitment continued until data saturation, defined as several consecutive interviews yielding no new themes relevant to the aim, and when all researchers independently agreed that the existing data adequately captured the range and depth of men’s experiences of localised UTI in PHC. At this point, further interviews were unlikely to add conceptual value, and data collection was concluded.

The interviews were audio-recorded and then transcribed verbatim by a professional transcriber.

Three researchers, two GPs (HKI, MT) and one social scientist (HG) read all transcripts repeatedly. The authors met regularly to read, code and discuss the different themes that emerged from the interviews. Once no new themes or insights emerged from the interviews as agreed from all researchers, the data were found to be saturated and the interviews were stopped. A thematic analysis was conducted with an inductive approach, to construct and analyze patterns and themes within the data systematically. An inductive coding, with empirical codes was first performed (supplement 2). Second, these codes were clustered into broader more abstract themes. Through analysis and discussions between the three researchers, the codes and meanings were condensed and finally synthesized as descriptions and concepts in the final themes [15].

### The authors’ backgrounds and prior understanding

The junior doctor had formal training on qualitative research methods but limited prior qualitative research experience. HKI who supervised the junior doctor has experience from research on infections in primary care including qualitative methods. MT, MA and HG all have lead qualitative studies and formal training. HG is a sociologist, non-clinical qualitative methodologist who added sociological aspects to the analysis and challenged any clinical biases.

### Ethical approval

Ethical approval was obtained from the Swedish Ethical Review Authority (Dnr 2022-01647-01). All participants gave informed consent before taking part.

## Results

The median age of the 18 patients was 77.5 years (61–84 years). Nine of the interviews were performed at a PHCC, six by telephone, and three in the patient’s home. The interviews lasted between 10 and 32 min. Among the 18 patients interviewed, 11 had an earlier or ongoing contact with a urologist. Six of the patients had intermittent catheterization.

The most frequent symptoms described in this study were urgency, frequent and painful micturition. Other symptoms described were frequent nocturia, localised abdominal pain, haematuria and foul-smelling urine, discomfort/burning sensation around the urethra.

**Figure 1.**
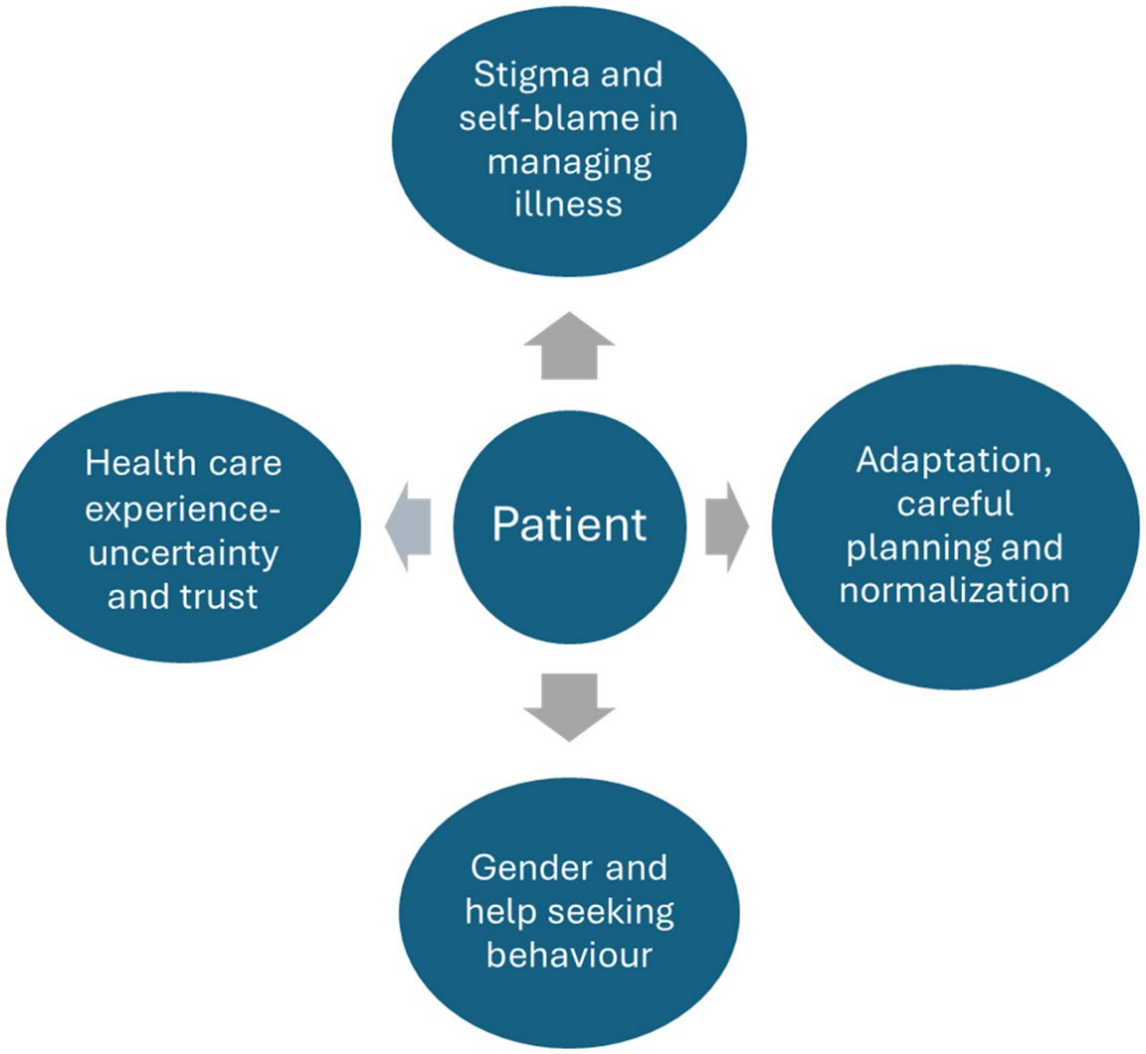
Four themes emerging from the interviews.

The four themes that emerged from the interpretation of the interviews were (1) stigma and self-blame in managing illness. (2) Adaptation, careful planning and normalization. (3) Gender and help seeking behaviour (4) Health care experience – uncertainty and trust (Figure 1).

### Stigma and self-blame in managing illness

When asked to describe the experience of their latest UTI, the men in this study often started their story with extended illness narratives that provided contextual aetiologies. They seemed to want to give the interviewer many details regarding the situation when they fell ill that might give clues as to why they were affected. They described the circumstances when their symptoms started.

So it was a Saturday…so I was at the out-of-hours clinic…It started on a Friday evening, you can say, and then I called them when I realized what it was. (65 years)

I had not done anything in particular, I was not dressed differently…. (61 years)

Most patients in our interviews reflected on the causes behind their localised UTI. In some of the interviews, there were aspects of self-responsibility and stigma adjacent self-blame and a sense of personal responsibility of illness. Worries of not having dressed warmly enough, getting too cold while swimming in a pool, or not being hygienic enough, a pattern common in moralized risk talk. Getting cold was the most commonly believed cause behind the onset of symptoms described by the patients.

“And why did you get it you wonder but I think I have been outdoors and become cold.” Now we sat outdoors yesterday, now I feel a little, I feel a little as soon as I’ve been outdoors and been cold. (65 years)

I tried swimming in the sea once last summer. I bathe a lot otherwise….Yes, I immediately felt it coming back. (62 years)

Most of the patients explained that they already suspected a LUTI when seeking care. Other patients felt unsure about their symptoms and searched for information elsewhere before they contacted their PHC.

But I thought it felt like a urinary tract infection, that is, as you have heard them say…. Plus you google it, of course… And then it said that it could probably…or it could be a urinary tract infection. (61 years)

so I didn’t know, but I think…I guess I have a…I feel that I have some understanding of what happens in a human body and so on, so I believed roughly what it was…. (67 years)

The patients reasoned about other explanations behind the localised UTI symptoms including age-related explanations and behavioral and expressed worries regarding underlying causes.

But that’s because I have a too big prostate. (76 years)

My wife claims that I drink too little (water)…then I also have problems with the prostate misery, I don’t want to drink too much…. (80 years)

Other explanations behind the UTI symptoms as fear of cancer were seldom expressed by the patients suggesting bounded worry within a common-sense model of illness representations.

### Adaptation, careful planning and normalization

The interviewed patients described various ways of adapting to the symptoms caused by the infection, including careful planning and noting the locations of public restrooms. Some normalized frequency and urgency as part of getting old, aligning with sociocultural aging narratives. Humor appeared as a coping resource and masculinity performance, for example, when symptoms were described such as urgency and frequent micturition. One patient found himself having visited the women’s restroom because he was in such a hurry. Many of the patients in this study described that the localised UTI symptoms affected their daily lives. Frequent micturition during nighttime led to disturbed sleep. Others refrained from swimming in the sea to avoid catching a new infection. The most common situation described was how urgency made them look out for available toilets in their vicinity and sometimes prevented them from leaving their home.

When it’s a hurry, sometimes it’s very much a hurry. (67 years)

Yes, when it’s at its worst…when you’re out, but I know where all the toilets are in the city. …laughter. (76 years)

Some noted an unpleasant smell due to their LUTI that made them avoid visiting toilets outside their home.

When you have an infection like that, you don’t want to go to the toilet anywhere else but at home. (68 years)

The men themselves described habituation and accommodation to the symptoms of LUTI and very often adjusted their way of living according to them.

It’s still pretty good, but you have to stay at home. It limits you, so to speak, it does. (65 years)

No, I haven’t had to, but I’ve had office work, so I’ve only had to run to the toilet, otherwise it has worked…it has been a bit rushed at some point every now and then to find a toilet. (67 years)

The men in this study seemed to regard frequent micturition as a common phenomenon in older men as they get older and therefore tend to normalize the symptoms of localised UTI. They found different solutions to handle the problem but also compared their symptoms with other male friends of the same age.

…I seldom sleep more than two hours before I have to get up and pee at night, so urgency I have it always, but I guess it is associated with the size of my prostate. (80 years)

### Gender and help seeking behaviour

The interviewed patients shared various aspects of their healthcare experiences and often described influence of gendered social support in their help-seeking. Some men explained that a female relative had encouraged them to seek medical attention for their symptoms.

Some of the interviewed men explained that they did not seek care immediately but rather postponed their contact with health care until a female relative (wife or daughter) insisted that they seek care.

So then, some time passed and so…I talked to my daughter about it, yes, but, dad, you have to make sure to see the doctor as soon as possible. (78 years)

I was sick and tired of the pain, and then I have a wife who chases after me, so that…. (81 years)

Some of the patients in the study experienced frustration. Especially, when having recurrent problems. And so, some patients positioned themselves by telling the healthcare personnel about relatives who have knowledge about the disease and how they followed their advice instead.

I have a wife who used to be a nurse so I suspected myself that it was a urinary tract infection. (82 years)

I talked to my wife, she’s a doctor herself…And she said, she’s retired, of course, but just go to the health care center and say; I think I have a urinary tract infection. (78 years)

### Health care experience – uncertainty and trust

The majority of the patients in this study expressed that their symptoms were taken seriously by healthcare personnel, and they were booked for a medical appointment the same day. However, some patients found the care stereotype particularly if their symptoms deviated from typical localised UTI symptoms. In these cases, they experienced difficulties accessing appropriate care and felt that their individual medical history was not fully considered. They also said they felt uninformed and insecure regarding their diagnosis. Certain patients described recurrent problems with fast deterioration in their localised UTI but found little understanding from health care personnel. Together, these experiences reflect concerns about person centredness and continuity in the diagnostic and treatment process.

It was good, but maybe…a little…what should I say…routinely, because if you had gone back and looked a bit at earlier symptoms, you might have prescribed a different type of antibiotic or for a longer period. (84 years)

Some said that they never had a proper explanation of their symptoms and so they were left in uncertainty regarding their medical condition.

And then I have taken a hell of a lot of blood samples…PSA and a couple of other samples and liver and kidneys and such things. I still don’t have an answer to why this happened and why it has been going on for such a long time as it did. (62 years)

One man described his contact with the triage nurse that said that localised UTI in men is so rare that it is unlikely to be a localised UTI:

But then I was a little confused when she said no, you can’t have that, but…yes, you could. (61 years)

“Yeah, I just thought what the hell it could be. …then you get a little more…then you think a little bit about prostate cancer or…”… So then I actually became…then I got a little worried. (61 years)

Some patients expressed frustration over fragmented care and weak communication between doctors with different specialties. In contrast, other patients expressed great confidence in the healthcare system. In general, the patients in this study were convinced that if they took the medicine they were prescribed all would end well.

…before there used to be doctors, that were at the hospital and they talked to each other and so on. (63 years)

So far there have been no problems with that matter, the doctor certainly knows his job, so he or she prescribes the right medicine, I am convinced of that. (82 years)

…if I have received the medicine and I take it, then I don’t think there is anything to worry about. (82 years)

Few of the patients in this study expressed worries about complications and the risk of more serious infections. They expressed great trust in their GPs and the medical care they could offer.

## Discussion

This study has explored expectations, experiences and symptoms in men with localised UTI in PHC. The interviewed men commonly sought explanations for localised UTI, frequently invoking self-blame or relying on common misconceptions like getting cold; they adapted their daily lives around symptoms while also normalizing them as age-related. Female relatives often facilitated timely help-seeking. Healthcare was generally accessible but some men perceived limited individualisation and insufficient explanation.

### Comparison with existing literature

We could not find any study that focused only on men with localised UTI in PHC with a qualitative approach. However, in a systematic review of qualitative research on UTI symptoms by Izett-Kay et al., two of 16 studies included male patients [13]. The two studies aimed to describe symptoms and treatment outcome among patients with intermittent catheter use [18,19]. Patients (men and women) described that they were unsure about which symptoms should prompt a visit to the PHCC, and when to seek care. Many of the symptoms presented were unspecific such as cloudy and foul-smelling urine [18]. These are symptoms also presented among patients in our study. Above all, inexperienced patients wanted to understand the reason behind their symptoms.

Many patients in our study expressed the view that onset of localised UTI symptoms was triggered by getting cold. A review of the literature revealed no empirical evidence supporting this belief. In unofficial websites with patient information about localised UTI, contradictory messages were found. Swedish official web pages with patient information do not mention the correlation between getting cold and developing a localised UTI [20]. On the contrary, there is some evidence for seasonal variation of localised UTI consultations in PHC where patients under 70 years have a September to November peak. This seasonal variation was not seen in patients above 85 years [21]. The causes of the peak in localised UTI incidence during the autumn in those aged 14–69 are not known.

In the systematic review [13] based on interviews with almost exclusively women, the women describe self-responsibility that they might have done something to precipitate the UTI episode and feel guilty about becoming infected. This corresponds with the feelings expressed by the interviewed men in our study. The men in our study expressed discomfort and self-judgement related to the feeling of being dirty and smelly when having a UTI.

In this study, the selection criterion was a diagnosis of localised UTI within the last six months. We did not ask about comorbidities. Some of the interviewed patients described, in addition to their acute localised UTI, symptoms that likely could be diagnosed as lower urinary tract symptoms (LUTS). LUTS in men represent a group of symptoms including storage, voiding and post-micturition symptoms affecting the lower urinary tract [22]. Age is a risk factor for LUTS that can occur in up to 30% of men older than 65 years of age [23]. LUTS is significantly more common than localised UTI in men in PHC and symptoms caused by localised UTI can be similar to symptoms caused by LUTS.

Qualitative studies performed among patients with LUTS suggest that many patients seeking care are worried about prostate cancer [24], which was not the case in our study where only a few expressed concerns regarding prostate cancer. In studies regarding LUTS one of the primary factors in seeking care was to rule out prostate cancer as a cause behind LUTS symptoms [25]. The more acute onset of symptoms in localised UTI could be one explanation for the difference in patients’ concerns regarding underlying causes. Long-standing messages linking urinary symptoms to the prostate have led many men to assume that LUTS might signal cancer, despite evidence that they do not [26]. This contributes to unnecessary worry during acute urinary symptoms. The way health care providers communicate with patients is important and affects the patients’ perceptions regarding symptoms. When the health care provider does not confirm the patient’s suspicion of localised UTI, the patient might start to fear other more serious diagnoses.

GPs commonly experience diagnostic uncertainty when assessing urinary symptoms in men. Male UTIs are perceived as uncommon and difficult to identify clinically, often requiring extra tests. Qualitative studies from France, Sweden and Ireland [8,9,27], describe male localised UTI as complex and ambiguous, with GPs feeling less experienced, unsure about appropriate investigations, and concerned about underlying conditions. These factors might explain why GPs sometimes struggle to validate patients’ interpretations and why patients perceive care as inconsistent or insufficiently individualised.

Several patients in our study expressed the view that the acute symptoms from the localised UTI affected the quality of their everyday life. They found it hard to leave home since they needed to be close to a toilet. For some patients, this led to isolation at home. These findings are in line with previous research [13,25,28,29]. Some patients describe sleep disturbances due to frequent micturition at night. In a qualitative study including patients with LUTS, it is described, as well as in our study, that frequent micturition during the day- and nighttime is considered by both patients and GPs to be a part of the normal aging process in men [30].

Inexperienced patients often asked relatives and other sources for advice prior to their healthcare visit. It seems to be a strategy particularly for male patients to prepare for the contact with healthcare. This is consistent with previous research. In a systematic review exploring barriers and facilitators to help-seeking behaviour, the researchers describe that men often face substantial barriers to seeking help for abnormal urinary symptoms, partly due to limited awareness of when and how to seek care. These challenges are even more pronounced among men from minority backgrounds, who may encounter additional cultural, linguistic or structural obstacles [31]. Further, research shows that traditional masculine norms discourage men from seeking help for health problems, as doing so may be seen as a sign of weakness [32].

Some of the men in our study described that a female relative advised them to seek care. This has also been described in other studies [33,34]. One qualitative study described that men avoid being labelled as ‘inappropriate attenders’ by saying that they have been ordered by their wife to contact health care. In the study, this phenomenon is described as a strategy that enables men to preserve their masculine image in a situation where it is threatened [33]. Experienced patients recognized their symptoms from previous episodes and knew that they had a UTI [11].

For some patients, the care was experienced as overly standardized and not adequately responsive to individual circumstances. Such a finding was also described in the systematic review by Izett-Kay [13] where some patients felt that they were not being listened to or taken seriously. The importance of being listened to was emphasized by patients in a Dutch study including only women with UTI in PHC [11].

### Strengths

To our knowledge, this is the first qualitative study to explore male patients’ views and experiences of localised UTI in PHC. We interviewed patients with recent experience from localised UTI to get accurate information and avoid recall bias. Patients originating from both rural and urban areas were represented. The median age of the interviewed patients was high, as could be expected, as localised UTI is more common in older men. The qualitative method gives us detailed information about patients’ thoughts and experiences and highlights information that can be hard to produce by other research methods. Three different researchers read and interpreted the interviews, of which one researcher is a social scientist who could bring in aspects other than medical points of view. The researchers independently read and coded the transcripts, then compared and discussed their interpretations. After initial independent coding, the research team met regularly to compare interpretations. Discrepancies were discussed in depth until a shared understanding was reached. Although we did not conduct formal member checking, we ensured that our analysis remained grounded in participants’ narratives by continuously referring to the data. Reflexive discussions within the team also supported a transparent and balanced interpretation process.

### Weaknesses

With a median age of 77.5 years, the findings mainly reflect older men’s experiences and may not generalize to younger men. Due to ethical regulations, the researchers were not allowed to directly address patients to inform them about the study. Instead, PHCC managers identified eligible individuals and distributed study invitations. This recruitment pathway may have introduced selection bias and has implications for transferability. This likely favoured participation from individuals who were more motivated, more affected by their symptoms, or more comfortable engaging with written communication. Men with milder symptoms or lower healthcare engagement may therefore be underrepresented. As all participants were Swedish-speaking, perspectives of men with limited language proficiency or different cultural backgrounds are absent, restricting transferability to broader PHC populations.

We did not ask about the patients’ medical history. Patients’ experiences of their infection and the care could be affected by other underlying conditions. A clearer interpretation of the men’s narratives requires distinguishing between localised UTI and LUTS. localised UTI is typically an acute infectious episode characterised by sudden onset of dysuria, frequency, urgency and suprapubic discomfort, whereas LUTS represents a chronic, multifactorial symptom complex involving storage, voiding and post-micturition symptoms. Although clinically distinct, the two syndromes overlap substantially in their symptom presentation. This overlap is particularly relevant in our sample, where one-third used intermittent catheters and many had prior urology contact, making it likely that some reported symptoms reflected underlying LUTS rather than localised UTI alone. This distinction is important because LUTS requires broader assessment and has different management pathways than localised UTI. The conflation of the two conditions may therefore contribute both to diagnostic uncertainty in PHC and to patients’ uncertainty about the cause and meaning of their symptoms. Because our study did not include stratified analyses, we cannot determine how experiences differed between men with and without prior urological conditions. Future research using subgroup comparisons would help clarify these differences.

The interviewers’ backgrounds and identities may have influenced the interviews. One interviewer was a female GP with experience in primary care, and the other was a female junior doctor. Their clinical background may have influenced how comfortable participants felt sharing their symptoms or concerns, and their gender may have shaped the ease and style of the conversation.

The context of data collection may have influenced participants’ willingness to disclose personal experiences. Telephone interviews can sometimes facilitate openness by providing greater anonymity, whereas interviews conducted at home or at the PHCC may create different levels of comfort or formality. These contextual differences could have affected the depth and style of participants’ narratives.

### Implications for research and practice

The belief that swimming in cold water and getting cold causes localised UTI is common, although there is no evidence for this causality. GPs should be perceptive regarding any such beliefs among patients with LUTI, as well as any self-imposed guilt that could lead to unnecessary lifestyle changes.

Our findings suggest that it is important to pay careful attention to these patients both in the nurse triage as well as in the doctors’ meeting, as the localised UTI symptoms very often affect their daily lives to a great extent.

Although the patients in this study expressed mostly good confidence in health care, they also found the care to not be individualized enough regarding adaptation to their previous medical history.

## Supplementary Material

FIG1.pptx

Participant information and consent form_English.docx

Supplement_Patient_Interview_guide.docx

Supplement2_Code tree.docx

## Data Availability

The data that support the findings of this study are available from the corresponding author upon reasonable request.
