# Supplementary figures and images for: Experiences among men with localised urinary tract infection in primary care: a qualitative study
Source: Scand J Prim Health Care. 2026 Mar 29;44(1):2647002. doi: 10.1080/02813432.2026.2647002 (PMC13037200; doi:10.1080/02813432.2026.2647002)

## Slide 1
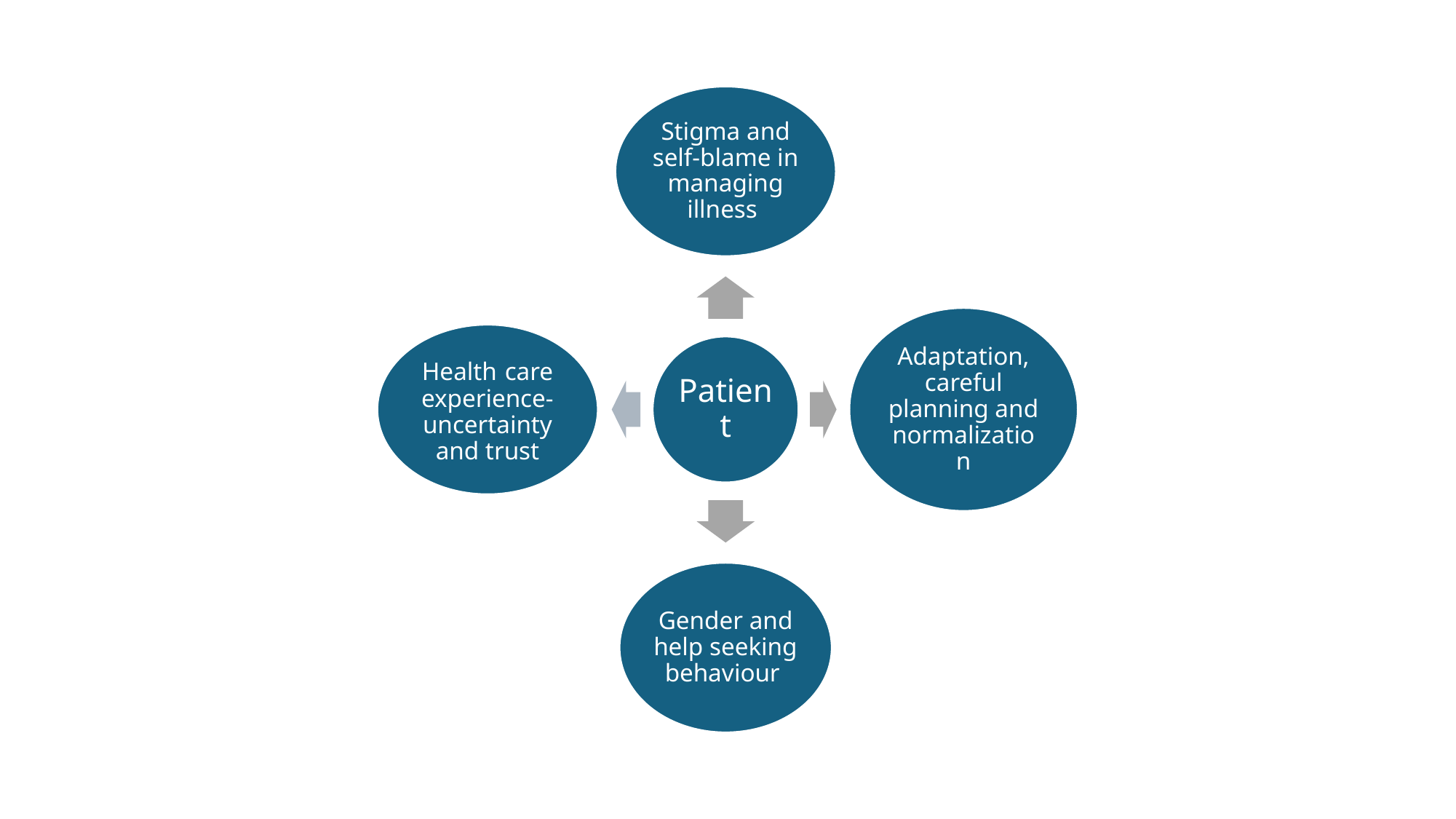

Supplement: FIG1.pptx [file IPRI_A_2647002_SM4210.pptx]
