# Supplementary material for: Experiences among men with localised urinary tract infection in primary care: a qualitative study
Source: Scand J Prim Health Care. 2026 Mar 29;44(1):2647002. doi: 10.1080/02813432.2026.2647002 (PMC13037200; doi:10.1080/02813432.2026.2647002)
Supplement: Participant information and consent form_English.docx [file IPRI_A_2647002_SM4209.docx]

**Research participant information and consent form:**

**Acute Cystitis in Primary Care**

Patient experiences of acute cystitis and expectations of treatment

**Information for Research Participants**

We would like to ask if you are willing to participate in a research project. This document provides information about the project and what participation involves.

**What is the project about, and why are you asking me to participate?**

The aim of this project is to learn more about men’s experiences of bladder infection (also referred to as acute cystitis). Bladder infections are a common diagnosis, often treated with different types of antibiotics. As part of efforts to improve antibiotic prescribing for bladder infections in men, we wish to gather insights from patients who have had this condition. It is important to evaluate patients’ experiences of symptoms and treatment to improve care if needed. What symptoms are associated with bladder infections? How is the treatment experienced? Through this study, we aim to better understand how men with bladder infections perceive their symptoms and treatment, with the goal of improving communication and care. You are being asked to participate because you are a man who has had a bladder infection within the past six months.

**How does the study work?**

If you fill in and submit the form below, we will contact you by telephone to provide more information about the study. We will invite you to an interview face to face or by telephone. During the interview, we will ask questions based on a structured questionnaire. We will ask about symptoms that may occur during a bladder infection, and you can describe whether you have experienced those symptoms and how bothersome they were. We will also ask about your experience with the treatment. The interview will take approximately 20 minutes. Following up on symptoms through an interview is different from routine care.

**Possible Risks or Disadvantages of Participating**

There are no risks associated with participating in the study. You always have the right to decline to answer any questions. You may also stop the interview at any time, without needing to provide a reason.

**What Happens to My Information?**

The project will collect and register information about you. The data we collect includes your age, gender, and the information you provide during the interview. Your responses will be handled confidentially so that no unauthorized person can access them. All printed transcripts will be stored securely in the same location.

Your personal data will only be stored in this secure database, and no one outside the research team will have access to it. Each participant will be assigned a study number. Only the anonymized transcripts with study numbers (not your personal details) will be used during analysis.

The purpose of processing your personal data is for scientific research. The data controller is Region Skåne. Under the EU General Data Protection Regulation (GDPR), you have the right to request access to your data, have any errors corrected, request deletion of your data, and restrict the processing of your data.

If you would like to access your data, please contact the responsible researchers (see contact info below).
You can also contact Region Skåne’s Data Protection Officer:
Dataskyddsombudet, Region Skåne
291 89 Kristianstad
Phone: 044–309 30 00
Email: region@skane.se

If you are dissatisfied with how your data is being processed, you have the right to lodge a complaint with the Swedish Authority for Privacy Protection (IMY).

**How Will I Learn About the Results?**

The results of the study will be published in a scientific journal.

**Insurance and Compensation**

You are covered by patient insurance. There is no financial compensation for participating in the study.

**Voluntary Participation**

Your participation is entirely voluntary, and you may withdraw at any time. If you choose not to participate or to withdraw, you do not need to provide a reason, and it will not affect your future care or treatment.

If you wish to withdraw, please contact the study coordinators (see below).

**Responsible Researcher**

Helena Kornfält Isberg,
Specialist in General Medicine,
Phone:
Email: helena.kornfaltisberg@skane.se

**Consent to Participate in the Study**

I have received both oral and written information about the study and have had the opportunity to ask questions. I will retain a copy of the written information.

☐ I consent to participate in the study: *Patient experiences of bladder Infection (acute cystitis) and expectations of treatment*

☐ I consent to the processing of my personal data as described in the participant information.

Place and Date: ____________________
Signature: ____________________
Printed Name: ____________________
