# Supplementary material for: Experiences among men with localised urinary tract infection in primary care: a qualitative study
Source: Scand J Prim Health Care. 2026 Mar 29;44(1):2647002. doi: 10.1080/02813432.2026.2647002 (PMC13037200; doi:10.1080/02813432.2026.2647002)
Supplement: Supplement_Patient_Interview_guide.docx [file IPRI_A_2647002_SM4208.docx]

Supplement 1

Interview guide, patient

**Information-introduction to the interview:**

My name is [interviewer’s name], and I will be conducting the interview today.

The purpose of this study is to explore patient experiences of bladder infections (acute cystitis) and their expectations of treatment. Thank you for agreeing to take part in the study. Before we begin, I’d like to ask if you have read the written information. Is everything clear, or do you have any questions?

Please note that neither you nor your healthcare center will be identifiable in any publications or presentations resulting from this study.

The interview will be audio recorded. The recording will be used only for transcription and analysis. Once the interviews are transcribed, the recordings will be stored securely and only accessible to the research team. They will not be shared with anyone outside the study.

You are free to withdraw from the interview at any time or skip any questions that you do not wish to answer, without needing to give a reason.

***Questions regarding the patient***: How old are you?

***Questions regarding UTI symptoms:***

-Please tell me about your latest episode of acute cystitis. (When? Where? How? Symptoms?)

-Did you experience acute cystitis more than once?

-How did the symptoms affect your everyday activities? (Did you have to stay home from work, how did you manage at home? Could you continue with your daily activities?)?

***Questions regarding health care seeking and treatment:***

-Can you describe your experience when you were seeking care? (What care and treatment did you get? What was your experience from your contact with health care?)

-What were your initial thoughts when seeking care? (What did you believe you needed help with?)

-Why did you decide to seek care?

-Did you have any particular worries regarding your symptoms/the infection?

- How did you experience the help and treatment you received?

-If you were prescribed antibiotics, how did you experience the treatment?

-Is there any information you would have liked to receive in connection with your infection?
