# Supplementary material for: Experiences among men with localised urinary tract infection in primary care: a qualitative study
Source: Scand J Prim Health Care. 2026 Mar 29;44(1):2647002. doi: 10.1080/02813432.2026.2647002 (PMC13037200; doi:10.1080/02813432.2026.2647002)
Supplement: Supplement2_Code tree.docx [file IPRI_A_2647002_SM4207.docx]

**Code tree**

**Theme 1: Stigma and self-blame in managing illness**

── Narrative framing and situational context

   └── Detailed introductions to the episode (setting, timing, circumstances)── Perceived causes and self‑infliction

│── Getting cold (outdoors, swimming)

── Insufficient warmth or clothing

   └── Hygiene concerns / not being clean enough

── Information‑seeking and lay knowledge

── Already suspected LUTI at care‑seeking

── Internet search (e.g., “I googled it”)

── Personal bodily understanding / self‑assessment

── Alternative explanations and differential reasoning

── Prostate enlargement / LUTS overlap

   └── Hydration beliefs (drinks too little; avoids drinking due to prostate issues)

└── Serious illness concern (infrequent)

    └── Occasional worry about cancer; generally rare or short‑lived

**Theme 2: Adaptation, careful planning and normalisation**

── Urgency management and toilet mapping

── Knows location of toilets in the city

└── Prevented from leaving home due to urgency

── Planning and lifestyle adjustment

── Careful planning to manage symptoms

  └── Staying at home as a strategy

── Activity avoidance

── Avoids cold exposure

└── Avoids bathing/swimming to prevent recurrence

── Odour and social embarrassment

  └── Avoids toilets outside home due to unpleasant urine odour

── Sleep disruption

└── Nocturia / frequent night‑time micturition driving disturbed sleep

── Work‑related functioning

   └── Office and self‑directed work are manageable (with frequent toilet breaks)

└── Social comparison, humour and normalisation

    ── Normalises frequent urination as common among older men

    ── Comical anecdotes (e.g., urgency leading to wrong restroom)

    └── Comparing symptoms with male peers / friends

**Theme 3: Gender and help seeking behaviour**

Influence of female relatives on help‑seeking and self‑care

── Encouragement by wife/daughter to seek care

── Use of wife’s urine dipsticks / home testing

── Reliance on relatives with medical training/knowledge

**Theme 4: Health care experience- uncertainty and trust**

── Access and responsiveness

── Same‑day appointment once PHC contacted; rapid access upon contact

── Standardised vs personalised care

── Care perceived as routine/stereotyped

── Limited individualisation; prior history not adequately considered

── Communication, explanation and uncertainty

── Lack of clear explanation for symptoms or recurrence

── Feeling uninformed and insecure about diagnosis── Continuity and coordination of care

  └── Frustration with poor communication across specialties / lack of continuity

── Trust and confidence in clinicians and treatment

── Great confidence in GP

└── Belief that taking prescribed medicine resolves the issue

── Triage and diagnostic gatekeeping

  ── Minimisation (e.g., “men rarely get LUTI”) causing confusion

    └── Transient worry triggered by contradicting triage messages (e.g., cancer)
